# Alzheimer’s Disease Severity Is Associated with an Imbalance in Serum Levels of Enzymes Regulating Plasmin Synthesis

**DOI:** 10.3390/ph15091074

**Published:** 2022-08-29

**Authors:** Francesco Angelucci, Katerina Veverova, Alžbeta Katonová, Lydia Piendel, Martin Vyhnalek, Jakub Hort

**Affiliations:** Memory Clinic, Department of Neurology, 2nd Faculty of Medicine, Charles University and Motol University Hospital, 150 06 Prague, Czech Republic

**Keywords:** Alzheimer’s disease, amnestic mild cognitive impairment, plasmin, tissue-type plasminogen activator, plasminogen activator inhibitor-1, ratio

## Abstract

Alzheimer’s disease (AD) is a central nervous system (CNS) disease characterized by loss of memory, cognitive functions, and neurodegeneration. Plasmin is an enzyme degrading many plasma proteins. In the CNS, plasmin may reduce the accumulation of beta amyloid (Aβ) and have other actions relevant to AD pathophysiology. Brain plasmin synthesis is regulated by two enzymes: one activating, the tissue plasminogen activator (tPA), and the other inhibiting, the plasminogen activator inhibitor-1 (PAI-1). We investigated the levels of tPA and PAI-1 in serum from 40 AD and 40 amnestic mild cognitively impaired (aMCI) patients compared to 10 cognitively healthy controls. Moreover, we also examined the PAI-1/tPA ratio in these patient groups. Venous blood was collected and the PAI-1 and tPA serum concentrations were quantified using sandwich ELISAs. The results showed that PAI-1 levels increased in AD and aMCI patients. This increase negatively correlated with cognitive performance measured using the Mini-Mental Status Exam (MMSE). Similarly, the ratio between tPA and PAI-1 gradually increases in aMCI and AD patients. This study demonstrates that AD and aMCI patients have altered PAI-1 serum levels and PAI-1/tPA ratio. Since these enzymes are CNS regulators of plasmin, PAI-1 serum levels could be a marker reflecting cognitive decline in AD.

## 1. Introduction

Alzheimer’s disease (AD) is a neurodegenerative disease of the central nervous system (CNS), characterized by the progressive loss of memory and other cognitive functions and by neuronal atrophy [1]. Investigation of its causes has led to various hypotheses, mainly based on the accumulation of beta amyloid (Aβ) and tau proteins in aggregates called amyloid plaques and neurofibrillary tangles [2]. Nonetheless, there are currently no effective treatments to halt the progression of the disease.

Newer hypotheses have been formulated, the most interesting concerns the role of soluble forms of Aβ, which could be the most direct cause of neuronal dysfunction and degeneration [3,4,5]. This hypothesis has led to a new understanding of disease mechanisms and potential therapeutic approaches. Among these mechanisms, we have recently drawn attention to the role of plasmin in AD pathogenesis [6].

Plasmin is an important hydrolase enzyme that degrades many proteins of the blood plasma, in particular, the protein fibrin [7]. Plasmin synthesis is regulated by the plasminogen activation system [8]. The human body does not directly produce plasmin, but its inactive precursor, plasminogen, which is activated by various substances. In the CNS, it has been observed that plasmin may have other functions that may be relevant to AD pathophysiology [9]. Plasmin is able to reduce the accumulation of Aβ by sequestering its soluble forms [10]. In addition, plasmin can activate NMDA receptors [11] and modulate the production of brain-derived neurotrophic factor (BDNF) [12], an essential factor for neuronal survival and synaptic activity [13]. Plasmin synthesis in the brain is regulated by two enzymes [14]: one activating, the tissue plasminogen activator (tPA), and the other inhibiting, the plasminogen activator inhibitor-1 (PAI-1) [15]. These enzymes can be produced by various elements of the CNS including neurons, astrocytes, and oligodendrocytes [14,16,17].

Many studies in humans have shown that AD patients can have alterations of these enzymes in the direction of a reduction in plasmin. Low levels of plasmin were observed in 67% of AD ApoE4 brains [18]. Furthermore, in two transgenic AD mouse models (Tg2576 and TgCRND8) it was observed that accumulation of Aβ peptide in the brain was associated with the upregulation of PAI-1 and inhibition of tPA [19]. In another study in post-mortem AD and control brain tissue [20], it was shown that both PAI-1 and tPA were elevated, thus, not altering plasmin synthesis, although the authors claim that increased tPA may cause synaptic plasticity, excitotoxic neuronal death, and apoptosis. In another study, plasmin and its precursor plasminogen were not altered in post-mortem AD versus control brain tissue [9]. However, reduced plasmin activity was observed in AD brains [9] indirectly favoring the accumulation of Aβ in aggregate or soluble forms [11].

Based on these data, we aimed at verifying whether the levels of tPA and PAI-1 in the serum of patients with AD dementia and with amnestic mild cognitive impairment (aMCI) due to AD are altered compared to healthy controls and whether they are associated with the severity of cognitive impairment. In addition, we examined whether the PAI-1/tPA ratio is altered in these patient groups. The ultimate goal was to verify whether the serum levels of these proteins could be useful from a diagnostic point of view to characterize the disease severity or to provide new therapeutic directions.

## 2. Results

### 2.1. Demographic Characteristics

Group demographic and clinical characteristics are reported in Table 1. There was no difference in sex distribution among the groups (*chi-square p value* = 0.297). There was a significant group effect for age, indicating that the control group was younger than the aMCI (*p* < 0.01) and AD (*p* < 0.01) groups. There was no difference between aMCI and AD groups (*p* = 0.713).

The AD and aMCI groups had significantly lower years of education as compared to the control group (*p* < 0.001), while there was no difference in education between aMCI and AD (*p* = 0.194). MMSE score was significantly lower in the aMCI (*p* < 0.001) and AD (*p* < 0.0001) groups as compared to controls. The AD dementia group also had a lower MMSE score compared to the aMCI group (*p* < 0.001).

### 2.2. Serum Levels of PAI-1 in AD, aMCI, and Controls

**Figure 1:**
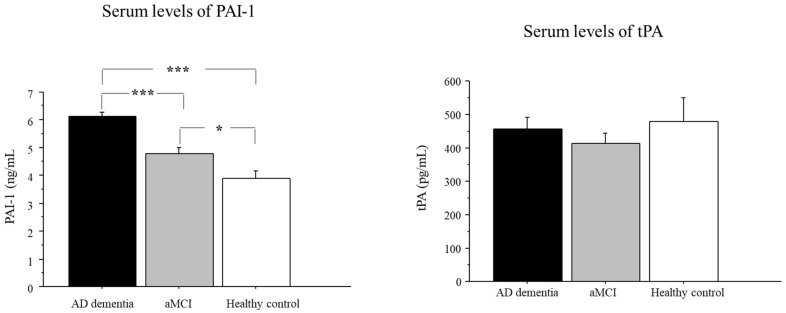
PAI-1 and tPA serum levels in AD dementia and aMCI patients and cognitively healthy controls. Data are the mean ± SEM. Values are expressed in ng/mL (PAI-1) and pg/mL (tPA). Asterisk (*) indicates a significant difference between the groups. * *p* < 0.05; *** *p* < 0.001.

Serum levels of PAI-1 in AD, aMCI, and controls are shown in Figure 1. There was a significant group effect in PAI-1 levels (*p* < 0.001). The post hoc analysis showed that PAI-1 levels were significantly higher in the AD group as compared to aMCI (*p* < 0.001) and controls (*p* < 0.001). Moreover, the aMCI group had significantly higher PAI-1 levels as compared to the control group (*p* < 0.05) (Figure 1).

### 2.3. Serum Levels of tPA in AD, aMCI, and Controls

Serum levels of tPA in AD, aMCI, and control groups are also shown in Figure 1. There was no significant group effect (*p* = 0.554). tPA serum levels did not significantly differ among the groups.

### 2.4. PAI-1/tPA Ratio in AD, aMCI, and Controls

**Figure 2:**
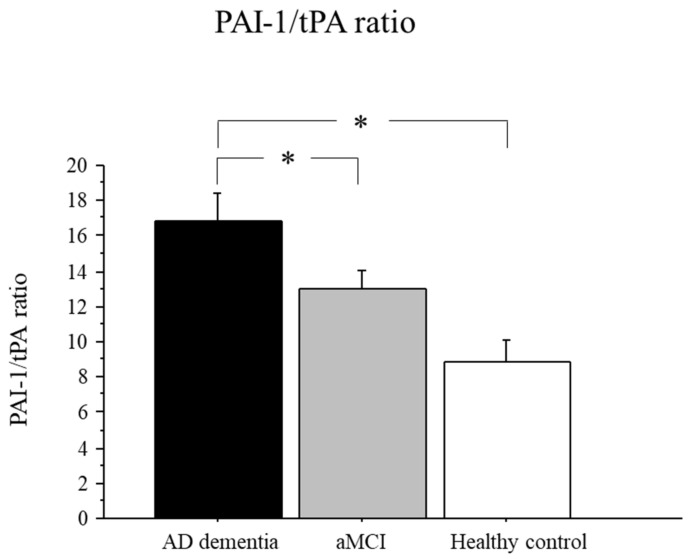
PAI-1/tPA ratio in AD dementia and aMCI patients and healthy controls. Data are the mean ± SEM. Values are expressed in pg/mL. Asterisk (*) indicates a significant difference between the groups. * *p* < 0.05.

The ratio between PAI-1/tPA serum levels is shown in Figure 2. There was a significant group effect (*p* < 0.05). Post hoc analysis showed that the PAI-1/tPA ratio was significantly higher in the AD group as compared to aMCI (*p* < 0.05) and controls (*p* < 0.01) (Figure 2).

An increased PAI-1/tPA ratio indicates that the balance between the levels of these enzymes in serum is altered in favor of an increase in PAI-1. In other words, while tPA levels are unchanged, PAI-1 levels are increased favoring an inhibition of plasmin synthesis.

### 2.5. Correlations between PAI-1/tPA Serum Levels and Disease Severity

**Figure 3:**
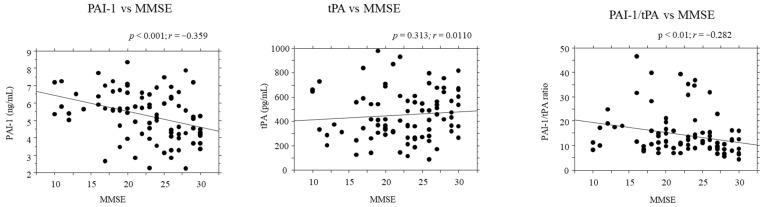
Correlations between serum levels of PAI-1, tPA, PAI-1/tPA ratio and disease severity (MMSE). *r* = Pearson correlation coefficient; *p* = *p* value.

The serum levels of PAI-1 negatively correlated with disease severity according to the MMSE score in the entire sample (*r* = −0.359, *p* < 0.001). In addition, the PAI-1/tPA ratio negatively correlated to the MMSE score (*r* = −0.257, *p* < 0.01). There was no significant correlation between tPA serum levels and MMSE score (Figure 3).

### 2.6. Correlations between PAI-1/tPA Serum Levels and Demographic Data

There was no significant correlation between the age of the subjects and PAI-1 (*r* = −0.016, *p* = 0.884) or tPA (*r* = 0.134, *p* = 0.232) serum levels. Similarly, the correlation between PAI-1/tPA ratio and age was not significant (*r* = −0.137, *p* = 0.220).

As for years of education, we did not find a significant correlation with PAI-1 (*r* = −0.173, *p* = 0.111), tPA (*r* = 0.080, *p* = 0.466), or PAI-1/tPA ratio (*r* = −0.096, *p* = 0.380).

## 3. Discussion

This study was performed to verify whether the serum levels of the enzymes regulating the synthesis of plasmin in CNS (tPA and PAI-1) are altered in patients with AD compared to cognitively healthy individuals. Another goal was to verify whether serum levels of these enzymes are correlated to the severity of cognitive deficit.

The results showed that, while tPA serum levels were unaffected, PAI-1 levels increased in patients with AD dementia and aMCI due to AD. This increase negatively correlated with cognitive performance measured by MMSE. In fact, PAI-1 levels were higher in AD patients than in aMCI patients and even more than in cognitively healthy controls. Similarly, but to a lesser extent, the ratio between tPA and PAI-1 gradually increases from controls to patients with aMCI and those with AD dementia.

These data suggest that in AD dementia, the synthesis of plasmin, and therefore its activity, may be either reduced or at least altered. This hypothesis is in line with other studies that demonstrate decreased plasmin activity in AD animal models and humans [9,19]. In animal models of AD [20], it has been observed that when PAI-1 is pharmacologically inhibited, plasmin increases its degrading activity on Aβ, an effect already observed in cell cultures [9,21,22].

In humans, it was shown that brain homogenates from AD patients had reduced plasmin activity and plasmin levels as compared to controls [19]. Our data suggest that the PAI-1 inhibitor may be the main cause of plasmin reduction. Supporting this notion, in similar studies it has been observed that PAI-1 plasma levels are increased in AD patients and that the levels correlate with the decline in cognitive functions [23,24].

A possible explanation comes from the fact that Aβ, especially in its soluble forms, can stimulate the production of PAI-1 by neurons and glial cells [25,26]. In animal models, it has been observed that this increase in PAI-1 occurs in the areas where Aβ accumulates [27,28] and the concomitant presence of inflammatory processes of microglia and astrocytes favor PAI-1 overproduction [29,30,31].

From a diagnostic point of view, the measurement of these enzymes in the blood, besides being a relatively simple procedure, could provide indications of the disease severity. The difference compared to other markers could be that these enzymes could indirectly indicate the presence and toxicity of soluble Aβ forms, and the degree of cognitive decline related to them.

The data obtained show that the serum levels of PAI-1 may work better for this purpose than the levels of tPA and the ratio between these two proteins. Indeed, the changes in PAI-1 levels are of greater magnitude as compared to those of the ratio PAI-1/tPA, while tPA levels are unchanged. However, it should be noted that in other studies tPA levels were also found to be altered in AD. From animal models of AD, it was observed that a decrease in tPA in the brain favors the accumulation of amyloid plaques [32,33]. In humans, the activity of tPA is reduced in the brains of AD patients [34] while the protein levels remain substantially unchanged [34,35]. These data suggest that a change in protein levels may not accompany a reduction in the enzymatic activity of tPA. The heterogeneity of the pathogenesis of AD in general and the complete clinical picture of each patient should also be considered. The presence of vascular diseases, for example, could affect the levels of these enzymes in the blood [36]. An encouraging fact is that the enzymes studied are responsible for the synthesis of plasmin in the CNS [14], and not only in the periphery [15]. At present, we cannot exclude that serum levels are not indicative of brain levels. Nonetheless, the negative correlation found between the serum levels of PAI-1 and the cognitive decline measured with MMSE is intriguing. The data on plasmin in AD could explain this correlation. If the synthesis or activity of plasmin is reduced in the brains of patients with dementia, the consequences could be Aβ accumulation [21], decreased synaptic plasticity [14], and therefore decreased cognitive functions [11,37]. Our research group is also currently testing the hypothesis that these enzymatic variations may be associated with an alteration in the turnover of BDNF between its preform and mature form [6,38]. In the case of a positive response, the association between the levels of these enzymes and BDNF could be another specific marker of cognitive performance. There are some limitations to our data interpretation. First, the number of healthy controls is relatively low, compared to that of the groups of patients. This is due to the difficulty of recruiting cognitive (and physiologically) healthy people in such types of studies. Another limitation is that in the present study we did not analyze other cognitive tasks such as memory and executive functions. For these reasons, our data on the possible use of PAI-1 as a biomarker must be considered as preliminary observations. Other studies in larger cohorts and analyzing other cognitive tasks as well as other variables must be carried out before reaching definitive conclusions.

## 4. Materials and Methods

### 4.1. Participants

Ninety participants from the database of the Czech Brain Aging Study, a longitudinal, memory clinic-based study on aging and cognitive impairment [39], were investigated. Of them, 40 participants were aMCI due to AD with high or intermediate likelihood of AD etiology [40], 40 were diagnosed with AD dementia with evidence of the AD pathophysiological process [41] and 10 were cognitively healthy participants. All participants underwent standard neurological and laboratory evaluations, comprehensive neuropsychological examination, and 1.5-T brain magnetic resonance imaging (MRI) within 3 months from the initial visit. All participants involved in this study signed written informed consent approved by the Motol University Hospital ethics committee.

### 4.2. Exclusion Criteria

The participants were excluded from the study if they had a history of neurological or psychiatric disorders other than AD potentially causing cognitive deficit (i.e., history of stroke, Parkinson disease, brain tumor, or alcohol abuse), hearing difficulties, depressive symptomatology (≥6 points on the 15-item Geriatric Depression Scale) [42] or had significant vascular impairment on the brain MRI (Fazekas scale more than 2) [43].

### 4.3. Blood Sampling

Venous blood was collected into sampling tubes that were centrifuged within 20 min after sampling at 2000× *g* for 20 min. Serum was then aliquoted and stored at −80 °C until analysis.

### 4.4. PAI-1 and tPA Determination

PAI-1 (Catalog Number: DY1786; R and D Systems, Minneapolis, MN, USA) and tPA (Catalog Number: DY7449; R and D Systems) serum levels were detected in sandwich ELISAs according to the manufacturer’s instructions. All assays were performed on F-bottom 96-well plates (Nunc, Wiesbaden, Germany). Tertiary antibodies were conjugated to horseradish peroxidase. Wells were developed with tetramethylbenzidine and measured at 450/570 nm. PAI-1 and tPA concentrations were quantified against a standard curve calibrated with known amounts of protein. Measurements were performed in duplicate and are expressed as ng/mL (PAI-1) and pg/mL (tPA).

### 4.5. PAI-1/tPA Ratio Determination

Values of serum PAI-1 were converted into pg/mL and used to calculate the PAI-1/tPA ratio according to the following formula: PAI-1 (pg/mL): tPA (pg/mL) = PAI-1/tPA ratio [24].

### 4.6. Statistical Analysis

Comparisons among the experimental groups (AD dementia, aMCI patients, and cognitively healthy controls) on PAI-1 and tPA serum levels were performed using univariate analyses of variance (ANOVA) followed by Fisher-protected least significant difference post hoc test. Categorical (nominal) data were analyzed using a *Chi-squared* test. Pearson correlation coefficients were calculated to explore relationships between biochemical and clinical/demographic data.

The level of statistical significance was set at *p* < 0.05. Statistical analysis was performed using the Statview software from the SAS Institute.

## 5. Conclusions

In conclusion, this study demonstrates that patients with AD dementia have altered serum levels of PAI-1 and an altered PAI-1/tPA ratio. Since these enzymes are CNS regulators of plasmin, serum levels of PAI-1 could have an important significance as a marker reflecting the underlying pathophysiology of cognitive decline. The association between these enzymatic levels and the levels of proteins involved in the regulation of synaptic activity, such as BDNF, is desirable. If confirmed and corroborated, these data could also open new therapeutic options based on the modulation of PAI-1/tPA in AD dementia patients. However, more studies are needed before reaching such a conclusion.

## Figures and Tables

**Table 1:** Clinical and demographic characteristics of AD, aMCI patients, and healthy subjects.

Parameter	AD Patients (*n* = 40)	aMCI Patients (*n* = 40)	Controls (*n* = 10)	Statistics
Age (years)	70.5 ± 9.44 *	69.8 ± 6.83 *	61.2 ± 12.4	* *p* < 0.01 vs. Controls
Sex (male/female)	12 M/28 F	17 M/23 F	2 M/8 F	*chi-square* *p* = 0.297
Years of education	13.5 ± 2.9 *	14.8 ± 3.1 *	17 ± 1.65	* *p* < 0.001 vs. Controls
MMSE	19 ± 3.84 **^,^^†^	25 ± 2.9 *	29.9 ± 0.33	* *p* < 0.001 vs. Controls ** *p* < 0.0001 vs. Controls ^†^ *p* < 0.001 vs. aMCI

Data are the mean ± standard deviation. *n* = number of subjects included in the study. AD = Alzheimer’s disease; aMCI = amnestic Mild Cognitive Impairment; M = male, F = female, MMSE = Mini Mental State Examination. * *p* < 0.001 vs. Controls; ** *p* < 0.0001 vs. Controls; ^†^ *p* < 0.001 vs. aMCI.

## Data Availability

Data is contained within the article.

## References

[1] Lane C.A., Hardy J., Schott J.M. (2018). Alzheimer’s disease. Eur. J. Neurol..

[2] Jagust W. (2018). Imaging the evolution and pathophysiology of Alzheimer disease. Nat. Rev. Neurosci..

[3] Counts S.E., He B., Prout J.G., Michalski B., Farotti L., Fahnestock M., Mufson E.J. (2016). Cerebrospinal Fluid proNGF: A Putative Biomarker for Early Alzheimer’s Disease. Curr. Alzheimer Res..

[4] Yang T., Li S., Xu H., Walsh D.M., Selkoe D.J. (2016). Large Soluble Oligomers of Amyloid β-Protein from Alzheimer Brain Are Far Less Neuroactive Than the Smaller Oligomers to Which They Dissociate. J. Neurosci..

[5] Alifragis P., Marsh J. (2018). Synaptic dysfunction in Alzheimer’s disease: The effects of amyloid beta on synaptic vesicle dynamics as a novel target for therapeutic intervention. Neural Regen. Res..

[6] Angelucci F., Čechová K., Průša R., Hort J. (2018). Amyloid beta soluble forms and plasminogen activation system in Alzheimer’s disease: Consequences on extracellular maturation of brain-derived neurotrophic factor and therapeutic implications. CNS Neurosci. Ther..

[7] Miszta A., Huskens D., Donkervoort D., Roberts M.J.M., Wolberg A.S., de Laat B. (2021). Assessing Plasmin Generation in Health and Disease. Int. J. Mol. Sci..

[8] Rosso M. (2008). Del The plasminogen activation system in inflammation. Front. Biosci..

[9] Barker R., Love S., Kehoe P.G. (2010). Plasminogen and plasmin in Alzheimer’s disease. Brain Res..

[10] Ledesma M.D., Da Silva J.S., Crassaerts K., Delacourte A., De Strooper B., Dotti C.G. (2000). Brain plasmin enhances APP α-cleavage and Aβ degradation and is reduced in Alzheimer’s disease brains. EMBO Rep..

[11] Nicole O., Docagne F., Ali C., Margaill I., Carmeliet P., MacKenzie E.T., Vivien D., Buisson A. (2001). The proteolytic activity of tissue-plasminogen activator enhances NMDA receptor-mediated signaling. Nat. Med..

[12] Gerenu G., Martisova E., Ferrero H., Carracedo M., Rantamäki T., Ramirez M.J., Gil-Bea F.J. (2017). Modulation of BDNF cleavage by plasminogen-activator inhibitor-1 contributes to Alzheimer’s neuropathology and cognitive deficits. Biochim. Biophys. Acta-Mol. Basis Dis..

[13] Leal G., Bramham C.R., Duarte C.B. (2017). BDNF and Hippocampal Synaptic Plasticity. Vitam. Horm..

[14] Samson A.L., Medcalf R.L. (2006). Tissue-Type Plasminogen Activator: A Multifaceted Modulator of Neurotransmission and Synaptic Plasticity. Neuron.

[15] Ploplis V., Castellino F. (2005). Structure and function of the plasminogen/plasmin system. Thromb. Haemost..

[16] Salles F.J., Strickland S. (2002). Localization and regulation of the tissue plasminogen activator-plasmin system in the hippocampus. J. Neurosci..

[17] Yepes M., Roussel B.D., Ali C., Vivien D. (2009). Tissue-type plasminogen activator in the ischemic brain: More than a thrombolytic. Trends Neurosci..

[18] Barker R., Kehoe P.G., Love S. (2012). Activators and inhibitors of the plasminogen system in Alzheimer’s disease. J. Cell. Mol. Med..

[19] Ledesma M.D., Abad-Rodriguez J., Galvan C., Biondi E., Navarro P., Delacourte A., Dingwall C., Dotti C.G. (2003). Raft disorganization leads to reduced plasmin activity in Alzheimer’s disease brains. EMBO Rep..

[20] Jacobsen J.S., Comery T.A., Martone R.L., Elokdah H., Crandall D.L., Oganesian A., Aschmies S., Kirksey Y., Gonzales C., Xu J. (2008). Enhanced clearance of A in brain by sustaining the plasmin proteolysis cascade. Proc. Natl. Acad. Sci. USA.

[21] Tucker H.M., Kihiko M., Caldwell J.N., Wright S., Kawarabayashi T., Price D., Walker D., Scheff S., McGillis J.P., Rydel R.E. (2000). The Plasmin System Is Induced by and Degrades Amyloid-β Aggregates. J. Neurosci..

[22] Tucker H.M., Kihiko-Ehmann M., Wright S., Rydel R.E., Estus S. (2002). Tissue Plasminogen Activator Requires Plasminogen to Modulate Amyloid-β Neurotoxicity and Deposition. J. Neurochem..

[23] Oh J., Lee H.-J., Song J.-H., Park S.I., Kim H. (2014). Plasminogen activator inhibitor-1 as an early potential diagnostic marker for Alzheimer’s disease. Exp. Gerontol..

[24] Wang J., Yuan Y., Cai R., Huang R., Tian S., Lin H., Guo D., Wang S. (2018). Association between Plasma Levels of PAI-1, tPA/PAI-1 Molar Ratio, and Mild Cognitive Impairment in Chinese Patients with Type 2 Diabetes Mellitus. J. Alzheimer’s Dis..

[25] Liu R.-M., van Groen T., Katre A., Cao D., Kadisha I., Ballinger C., Wang L., Carroll S.L., Li L. (2011). Knockout of plasminogen activator inhibitor 1 gene reduces amyloid beta peptide burden in a mouse model of Alzheimer’s disease. Neurobiol. Aging.

[26] Buisson A., Nicole O., Docagne F., Sartelet H., Mackenzie E.T., Vivien D. (1998). Up-regulation of a serine protease inhibitor in astrocytes mediates the neuroprotective activity of transforming growth factor β1. FASEB J..

[27] Cacquevel M., Launay S., Castel H., Benchenane K., Chéenne S., Buée L., Moons L., Delacourte A., Carmeliet P., Vivien D. (2007). Ageing and amyloid-beta peptide deposition contribute to an impaired brain tissue plasminogen activator activity by different mechanisms. Neurobiol. Dis..

[28] Melchor J.P., Pawlak R., Strickland S. (2003). The Tissue Plasminogen Activator-Plasminogen Proteolytic Cascade Accelerates Amyloid-β (Aβ) Degradation and Inhibits Aβ-Induced Neurodegeneration. J. Neurosci..

[29] Sawdey M.S., Loskutoff D.J. (1991). Regulation of murine type 1 plasminogen activator inhibitor gene expression in vivo. Tissue specificity and induction by lipopolysaccharide, tumor necrosis factor-alpha, and transforming growth factor-beta. J. Clin. Investig..

[30] Podor T.J., Joshua P., Butcher M., Seiffert D., Loskutoff D., Gauldie J. (1992). Accumulation of Type 1 Plasminogen Activator Inhibitor and Vitronectin at Sites of Cellular Necrosis and Inflammation. Ann. N. Y. Acad. Sci..

[31] Kosaka K., Iseki E., Hino H., Akiyama H., Kondo H., Kato M., Ikeda K. (2002). Immunohistochemical localization of plasminogen activator inhibitor-1 in rat and human brain tissues. Neurosci. Lett..

[32] Oh S.B., Byun C.J., Yun J.-H., Jo D.-G., Carmeliet P., Koh J.-Y., Lee J.-Y. (2014). Tissue plasminogen activator arrests Alzheimer’s disease pathogenesis. Neurobiol. Aging.

[33] Bi Oh S., Suh N., Kim I., Lee J.-Y.Y. (2015). Impacts of aging and amyloid-β deposition on plasminogen activators and plasminogen activator inhibitor-1 in the Tg2576 mouse model of Alzheimer’s disease. Brain Res..

[34] Fabbro S., Seeds N.W. (2009). Plasminogen activator activity is inhibited while neuroserpin is up-regulated in the Alzheimer disease brain. J. Neurochem..

[35] Medina M.G., Ledesma M.D., Domínguez J.E., Medina M., Zafra D., Alameda F., Dotti C.G., Navarro P. (2005). Tissue plasminogen activator mediates amyloid-induced neurotoxicity via Erk1/2 activation. EMBO J..

[36] Kanno Y. (2019). The Role of Fibrinolytic Regulators in Vascular Dysfunction of Systemic Sclerosis. Int. J. Mol. Sci..

[37] Huang Y.Y., Bach M.E., Lipp H.P., Zhuo M., Wolfer D.P., Hawkins R.D., Schoonjans L., Kandel E.R., Godfraind J.M., Mulligan R. (1996). Mice lacking the gene encoding tissue-type plasminogen activator show a selective interference with late-phase long-term potentiation in both Schaffer collateral and mossy fiber pathways. Proc. Natl. Acad. Sci. USA.

[38] Mossiat C., Prigent-Tessier A., Garnier P., Marie C., Jacquin A., Rodier M., Béjot Y. (2014). Exogenous t-PA Administration Increases Hippocampal Mature BDNF Levels. Plasmin- or NMDA-Dependent Mechanism?. PLoS ONE.

[39] Sheardova K., Vyhnalek M., Nedelska Z., Laczo J., Andel R., Marciniak R., Cerman J., Lerch O., Hort J. (2019). Czech Brain Aging Study (CBAS): Prospective Multicentre Cohort Study on Risk and Protective Factors for Dementia in the Czech Republic. BMJ Open.

[40] Albert M.S., DeKosky S.T., Dickson D., Dubois B., Feldman H.H., Fox N.C., Gamst A., Holtzman D.M., Jagust W.J., Petersen R.C. (2011). The diagnosis of mild cognitive impairment due to Alzheimer’s disease: Recommendations from the National Institute on Aging-Alzheimer’s Association workgroups on diagnostic guidelines for Alzheimer’s disease. Alzheimers. Dement..

[41] McKhann G.M., Knopman D.S., Chertkow H., Hyman B.T., Jack C.R., Kawas C.H., Klunk W.E., Koroshetz W.J., Manly J.J., Mayeux R. (2011). The diagnosis of dementia due to Alzheimer’s disease: Recommendations from the National Institute on Aging-Alzheimer’s Association workgroups on diagnostic guidelines for Alzheimer’s disease. Alzheimers. Dement..

[42] Yesavage J.A. (1988). Geriatric Depression Scale. Psychopharmacol. Bull..

[43] Fazekas F., Chawluk J., Alavi A., Hurtig H., Zimmerman R. (1987). MR signal abnormalities at 1.5 T in Alzheimer’s dementia and normal aging. Am. J. Roentgenol..

